# Foreign Correspondence

**Published:** 1840-09-19

**Authors:** 


### FOREIGN CORRESPONDENCE.

PARIS, 6*th August*, 1840.

To the Editors of the Medical Examiner.

We have already presented to our readers that portion of the essay of Messrs. ANDRAL and GAVARET, on the *variation of the elements of the blood in diseases*, read before the Academy of Sciences on the 27th ult., and which related to that class of affections in which the fibrine is found in excess. At the last session—August 3d—M. Andral continued the reading of his memoir, and treated of three other classes of diseases, which are characterized by different variations in the proportions of the elements of the blood.

The *second class* comprises those diseases in which the *fibrine* is in normal quantity, or is diminished, at the same time that the globules remain normal in quantity, or are increased. This class is divided into two orders:—1. Pyrexiæ, or Fevers. 2. Congestions, and Hæmorrhagies.

During the course of continued fever there is never augmentation, and frequently diminution of the fibrine, which is sometimes diminished one-thousandth. Neither is there decrease in the red globules before blood-letting, and they often amount to and even exceed 40. In their progress, should there be phlegmasial complications, what we have just said continues to hold good. In one case, where there was severe anginitis, the globules attained the enormous number of 185, and notwithstanding the fibrine maintained its natural proportions.

*Typhoid Fever.*—(By this our authors understand that form of continued fever which is accompanied with an exanthematous, and subsequently ulcerated state of the intestinal follicles.) In consequence of the phlegmasial alteration, which the intestine undergoes, and which forms the anatomical character of typhoid fever, it might naturally be supposed that in this disease, we ought to find the blood in the same condition as in other inflammations. But it is not so. Whatever may be the violence of the intestinal phlegmasia, the blood developes no traces of it. No matter at what stage of typhoid fever we examine the blood—and this examination has been made from the fourth to twenty-first day—you never find the fibrine exceeding its normal standard. It often preserves its balance, although sometimes falling hence to it, thus offering entirely an inverse condition to that which we found in the phlegmasiæ. Whilst in these last the fibrine increases in direct proportion to the intensity of the inflammation, in typhoid fever, on the contrary, the fibrine diminishes in direct ratio to the gravity of the affection, and its decrease at times is so great as to fall below one-thousandth. Typhoid is of all diseases the one in which the number of the fibrine attained its greatest diminution. As to the globules, whilst in the phlegmasiæ, we often find them with a number less elevated at the commencement of the disease; the reverse takes place in typhoid. The more we examine the blood at a period removed from the moment of invasion, the more you will find cases in which the globules only have not diminished, but in which they have notably increased. Thus, as far as the eighth day, it is not uncommon to find the number of the globules at 140 to 150; whilst in acute rheumatism, and in pneumonia, at the same period, they are rarely more elevated than 130. Further, at an epoch already far removed from the period of attack, in typhoid fever we constantly see, in spite of both blood-letting and diet, the globules maintain a number much above 130, a fact which does not occur in the phlegmasiæ. At the same time—and this is very remarkable—this elevated number may not have taken place, or may have ceased to exist, and yet typhoid fever nevertheless occurs, and its march is not less regular. There are, then, here many cases to distinguish,—and one is, that typhoid fever is a morbid condition infinitely more complex than a simple phlegmasia.

*Eruptive Fevers*—(Variola, varioloid, measles, scarlet fever.)—In this class of fevers the fibrine has been found at one, and has never exceeded four. This maximum was, however, only presented once.

It is without doubt astonishing that in a disease where, as in small-pox, the skin is the seat of an abundant suppuration, the blood, obedient to the law of phlegmasiæ, should not contradict our labours by an increase in the quantity of the fibrine. It is that the cutaneous phlegmasia, like the intestinal one, in typhoid fever, is but a simple element of a more general affection which controls it, and from which the blood receives its character. As to the globules, they have offered yet a considerable increase in many cases of scarlet fever and measles, rising, for example, to 146, and on the contrary, they have sensibly augmented in any case of variola.

*Intermittent Fevers.*—In all cases where the blood has been examined, whether it was drawn during the paroxysm or the apyrexia, nothing positive has resulted.

*Congestions and Cerebral Hæmorrhagies.*—In the majority of the cases, but *not* in all, the *fibrine* has been found below the normal standard, whilst the *globules* had preserved their normal mean, or had exceeded it, and this result was much more marked when the blood was examined at an epoch further removed from the period of invasion.

In the *third class*, MM. Andral and Garant comprise those diseases in which the *red globules of the blood are diminished.* They state a number of facts relative to different morbid states which are known by this condition of the blood. We shall only give the names of some, such as some of the dropsies, that peculiar morbid state which is the sequela of certain intermittent fevers, (the special cachexy presented by the workmen in lead manufactories,) reserving our space for some details on

*Chlorosis.*—In this disease there is a first degree which is so little characterized by external symptoms, that the young girls appear at first view plethoric; but it is a false plethora, which announces itself already by the state of the blood, for it already gives less globules than the natural state,—but the difference is yet little appreciable—it soon becomes more so. We then find in the blood a diminution in the red globules that is not found at this period in any other disease, except in accidental conditions, when profuse hæmorrhagies have exhausted the organism. In one of these last cases, cited in the preceding memoir, it has been seen how the number of the globules descended as low as 21; in chlorosis it has fallen from the mean number, 127, to 38,—more ordinarily it is about 50.

After the administration of iron in chlorosis for some time, on examining the blood we find a sensible increase in the globules. Thus, in one of the cases cited, it was seen, under the influence of this treatment, to rise rapidly from 46 to 95. As to the other components of the blood, (except the water, which increases in ratio to the diminution of the red globules,) they remain totally uninfluenced; the fibrine neither decreases with the progress of the disease, nor rises under the use of the martials. These observations, it should be understood, apply only to simple chloroses,—for if they should be complicated by a phlegmasia, this phlegmasia is discovered at once by an augmentation in the proportions of the fibrine.

In the *fourth class* are included those diseases where there is *a diminution in the albumen.* When the renal secretion becomes so modified that albumen is found in the urine, this element is found in diminished quantity in the blood. This result, already stated by different writers, has been verified by our authors; who have found not more in Bright's disease of albumen than 56 to 60, in place of 72, the mean. No other modification was discovered in the other elements of the blood, save such as were accidental. Thus, in one case, and acute phlegmasia supervening suddenly, instantly increased the quantity of blood. On the other hand, a continued regimen causes as great a decrease in the quantity of the globules.

Our authors concluded their memoir in these words:—"Thus the further we have advanced in our researches, the easier we have found, by an analysis of facts, to trace to certain principles the causes of all the changes in the composition of the blood, which, by their mobility, and the rapidity of the changes, would seem at first sight to escape all rule, and to be the results of chance. In the midst of this apparent disorder, there are laws which are fulfilled, and to find them it is necessary to disengage the phenomena from all complications."

